# Supplementary material for: Degree and site of chromosomal instability define its oncogenic potential
Source: Nat Commun. 2020 Mar 20;11:1501. doi: 10.1038/s41467-020-15279-9 (PMC7083897; doi:10.1038/s41467-020-15279-9)
Supplement: Supplementary file 1 — Supplementary Information [file 41467_2020_15279_MOESM1_ESM.pdf]

Supplementary Information

Degree and site of chromosomal instability define its oncogenic potential

Hoevenaar et al.

Hoevenaar et al., Supplementary Figure 1

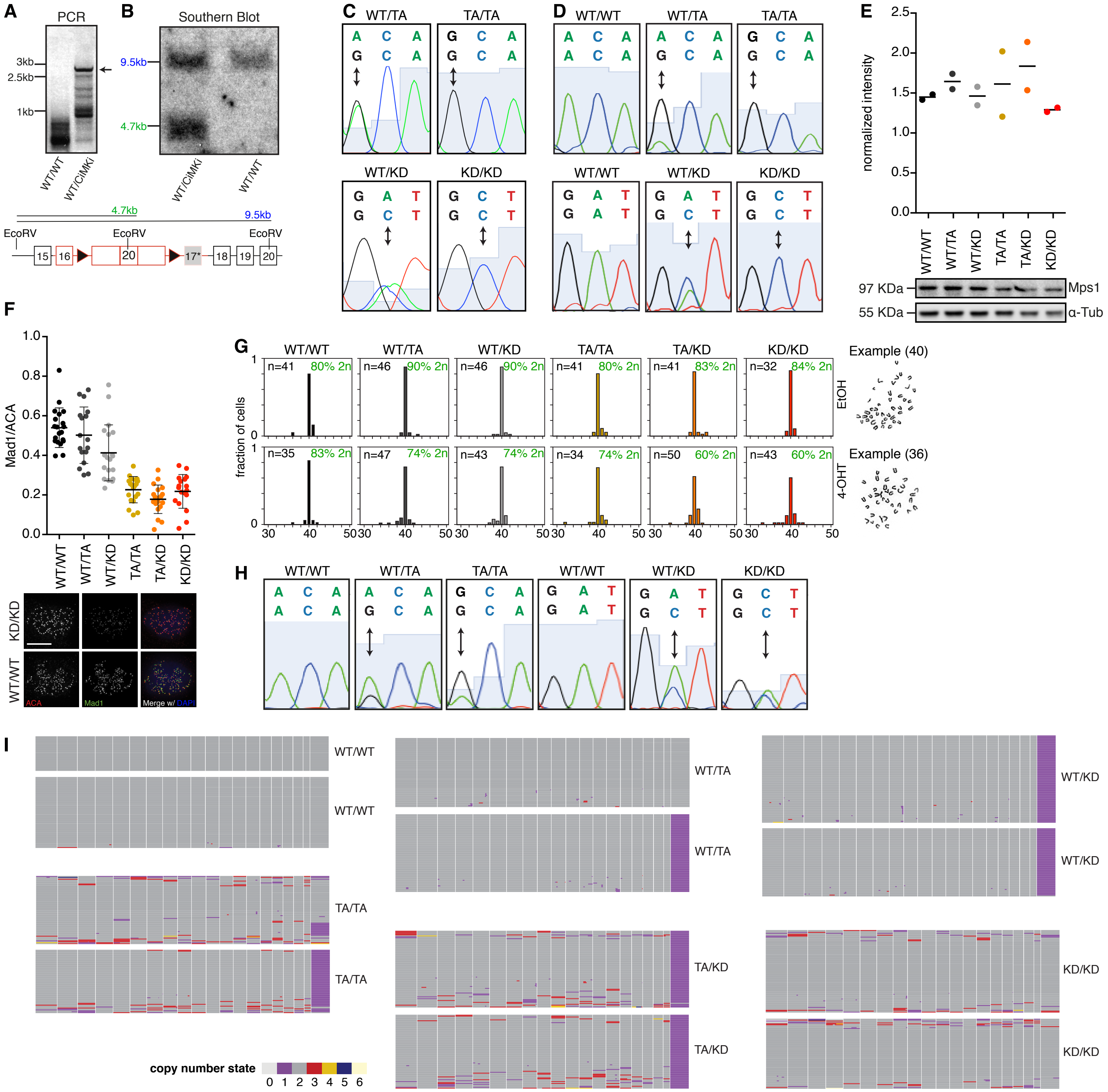

Supplementary Figure 1: A novel mouse model for CIN: Cre-inducible *Mps1* Knock-in (CiMKi). **(A)** Genomic PCR of targeted ES cells confirming presence of the *CiMKi* allele. Shown here specifically is the *CiMKi-T649A* ES clone that was used for blastocyst injection. **(B)** Confirmation of correct integration of the CiMKi alleles by Southern blot. Shown here specifically is the *CiMKi-T649A* ES clone that was used for blastocyst injection. Lower schematic shows EcoRV restriction sites used for Southern blot, indicated 5' of exon 15 and in exon 20. **(C)** Targeted sequencing confirming presence of *Mps1* mutations in mouse ear genomic DNA. **(D)** RT-PCR followed by targeted sequencing of cDNA from CiMKi MEF lines 56 hours after 4-OHT addition shows hetero- or homozygous expression of both D637A (A to C) or T649A (A to G) alleles. **(E)** MPS1 protein expression in *CiMKi;Rosa26-CreER^{T2}* MEFs 56 hours after 4-OHT addition as measured by Western blot (representative image). Intensity is normalized over α-tubulin and dot plot shows the average of two independent experiments. **(F)** Examples and quantification of Mad1 localization on kinetochores as a proxy for MPS1 activity in *CiMKi;Rosa26-CreER^{T2}* MEFs 72 hours after 4-OHT addition. Cells were blocked in mitosis by nocodazole and MG132 for 30 minutes. Graph shows quantifications of kinetochore signals as ratios over ACA signals. Data represents mean ± SD, n=20 (WT/WT, WT/KD, TA/TA, TA/KD), n=17 (WT/TA), n=18 (KD/KD) cells per condition. Scale bar 5 μm.

(continued on next page)

(G) Examples and quantification of diploid and aneuploid cells on metaphase spreads (DAPI) of *CiM-Ki;R26CreER^{T2}* primary MEFS 56 hours after 4-OHT addition. MEFs were blocked in mitosis by 4 hours treatment with nocodazole. Ploidy was assessed by counting the number of chromosomes per cell (n), percentage of diploid cells is given. **(H)** RT-PCR followed by targeted sequencing on cDNA from *CiM-Ki;R26CreER^{T2}* small intestine tissue one week after intraperitoneal tamoxifen injection confirms effective recombination and expression of the mutant alleles. Hetero- or homozygous expressions of both D637A (A to C) or T649A (A to G) alleles are shown. **(I)** scKaryo-seq (bin size 5 MB) showing ploidy in individual cells (horizontal lines) of small intestine 7 days after induction of the various CiMKi genotypes. Graphs show cells of two examples per genotype, one example per genotype and average aneuploidy and heterogeneity scores are shown in Fig. 1F. Colors indicate copy number state for a given chromosome. Source data for panels A, B, E-G are provided as a Source Data file.

Hoevenaar et al., Supplementary Figure 2

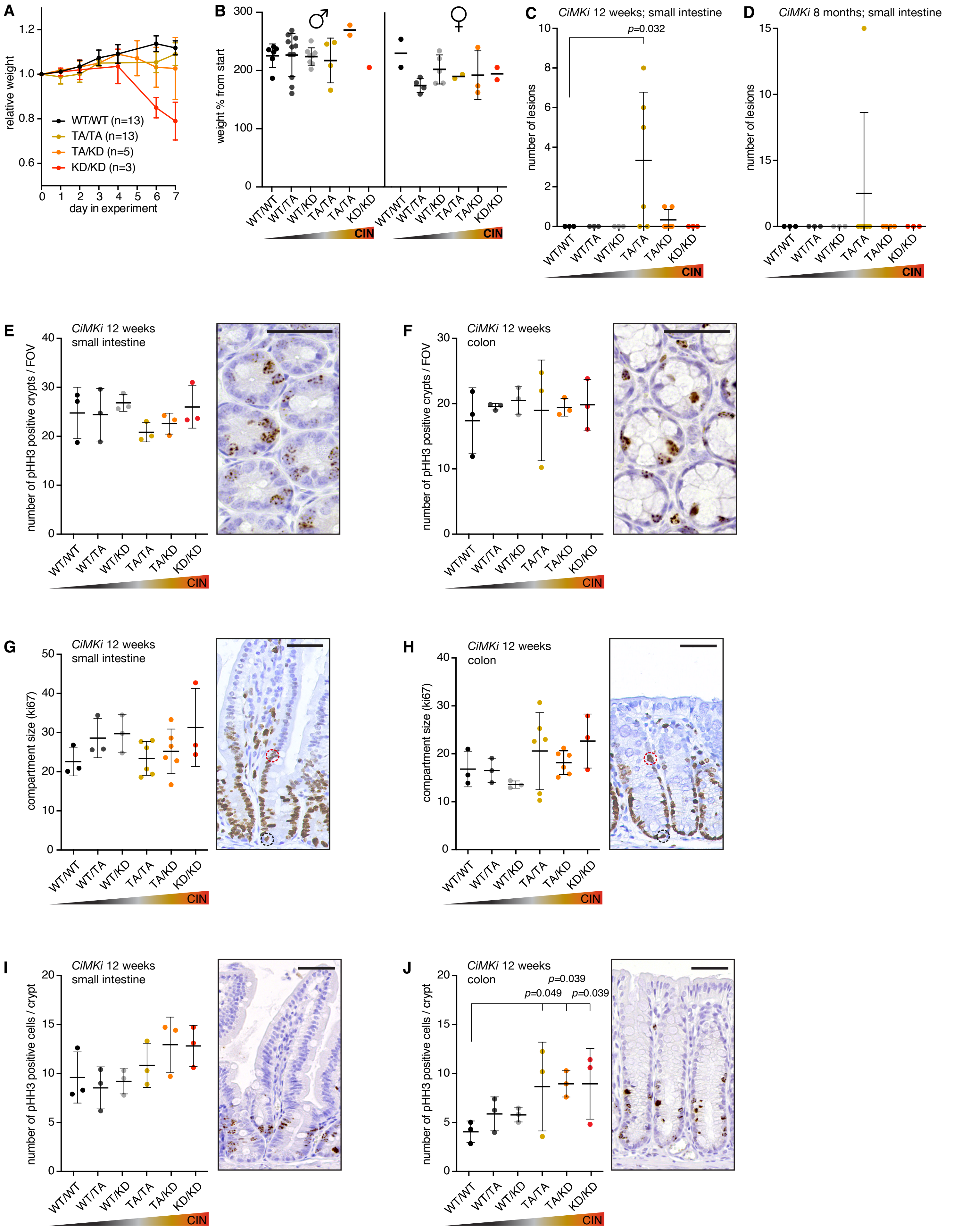

(continued on next page)

Hoevenaar et al., Supplementary Figure 2

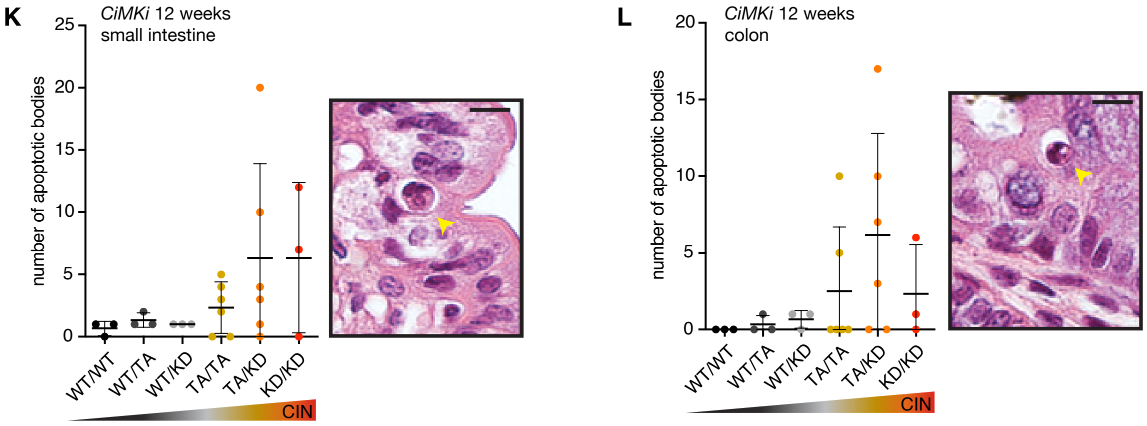

Supplementary Figure 2: Effects of CIN induction in the intestine

(A) Relative bodyweight of *CiMKi;R26CreER*^{T2} mice after three consecutive days of intraperitoneal tamoxifen injection. Lines represent change in bodyweight per group as fraction of their weight at the start of the experiment (mean ± SD). At day 7, mice from the *CiMKi*$^{TA/KD}$ and *CiMKi*$^{KD/KD}$ groups reached the humane endpoint and were euthanized. **(B)** Relative body weight in male (left; n=6 (WT/WT), n=11 (WT/TA), n=8 (WT/KD), n=4 (TA/TA), n=2 (TA/KD), n=1 (KD/KD) and female (right; n=2 (WT/WT), n=4 (WT/TA), n=5 (WT/KD), n=2 (TA/TA), n=3 (TA/KD), n=2 (KD/KD) *CiMKi;VillinCre* mice. Increase in weight is shown as percentage from start of the experiment (4 weeks) to end (8 months). Data is shown as mean percentage increase ± SD. **(C, D)** Quantifications of the number of adenomas as determined by H&E staining in small intestine of 12-week-old (C) and of 8-month-old (D) *CiMKi;VillinCre* mice. Data represents mean ± SD, n=3 (WT/WT, WT/TA, WT/KD, KD/KD), or n=6 (TA/TA, TA/KD) mice per genotype (C), n=3 (WT/WT, WT/TA, WT/KD, KD/KD), n=6 (TA/TA), or n=4 (TA/KD) mice per genotype (D), one-tailed Welch's t-test, comparing each group to *CiMKi*$^{WT/WT}$*VillinCre*; exact p-values are indicated when <0.05. In all groups, except for the moderate CIN groups, we observed normal mucosa of the small intestine and colon, and normal crypt villus ratio (1:3), brush border and epithelium lining consisting of goblet cells and enterocytes in the small intestine. Cellularity of the lamina propria was within normal limits, and scattered lymphoid aggregates were observed. In the colon, the crypts were regularly arranged and lined with goblet cells. The lamina propria contained normal cellularity, with scattered lymphoid aggregates. **(E, F)** Quantifications of the number of viable crypts of 12-week old *CiMKi;VillinCre* mice as determined by pHH3 positivity in small intestine (E) and colon (F). Data represent average numbers of 10 (40x) fields of view (FOV), and mean ± SD of 3 mice per genotype. Images are representatives for pHH3 staining. Scale bars 50 μm. **(G, H)** Proliferative compartment in small intestine (G) and colon (H) of 12-week old *CiMKi;VillinCre* mice as determined on ki67 stained tissue sections. Images show example crypts of small intestine and colon of *CiMKi;Apc*$^{Min/+}$*;VillinCre* mice (see Fig. 3 and 4) for clarification of scoring: compartment sizes were determined by scoring the number of cells between the first positive cell at the bottom of the crypt (black dotted circle) and the last positive cell in the transit amplifying zone (red dotted circle). Scale bars 50 μm. Dot plots show the average size of the compartment for each mouse (10 crypts (with normal appearance, selected from similar regions (~2/3 from proximal site) per mouse), and mean ± SD of 3 mice (WT/WT, WT/TA, WT/KD, KD/KD), or 6 mice (TA/TA, TA/KD) per genotype. **(I, J)** Quantifications of the number of mitotic cells as determined by pHH3 staining in small intestine (I) and colon (J). Dot plots show the average number of positive cells for each mouse (n=3 per genotype, 10 crypts (with normal appearance, selected from similar regions (~2/3 from proximal site) per mouse), and mean ± SD of 3 mice per genotype; ordinary one-way ANOVA, uncorrected Fisher's LSD test, exact p-values are indicated when <0.05. Images are representatives for pHH3 staining. Scale bars 50 μm. **(K, L)** Quantifications of the number of apoptotic bodies in H&E stained sections of small intestine (K) and colon (L). Data represent average numbers of 25 normal appearing (longitudinally oriented) crypts, and mean ± SD of 3 mice (WT/WT, WT/TA, WT/KD, KD/KD), or 6 mice (TA/TA, TA/KD) per genotype. Apoptotic bodies were recognized according to strict morphological criteria such as cell shrinkage with retracted pink to orange cytoplasm, chromatin condensation and nuclear fragmentation and separation of cells by a halo from adjacent enterocytes. Images show examples of apoptotic bodies (yellow arrow heads). Scale bars 10 μm. Source data for panels A-L are provided as a Source Data file.

Hoevenaar et al., Supplementary Figure 3

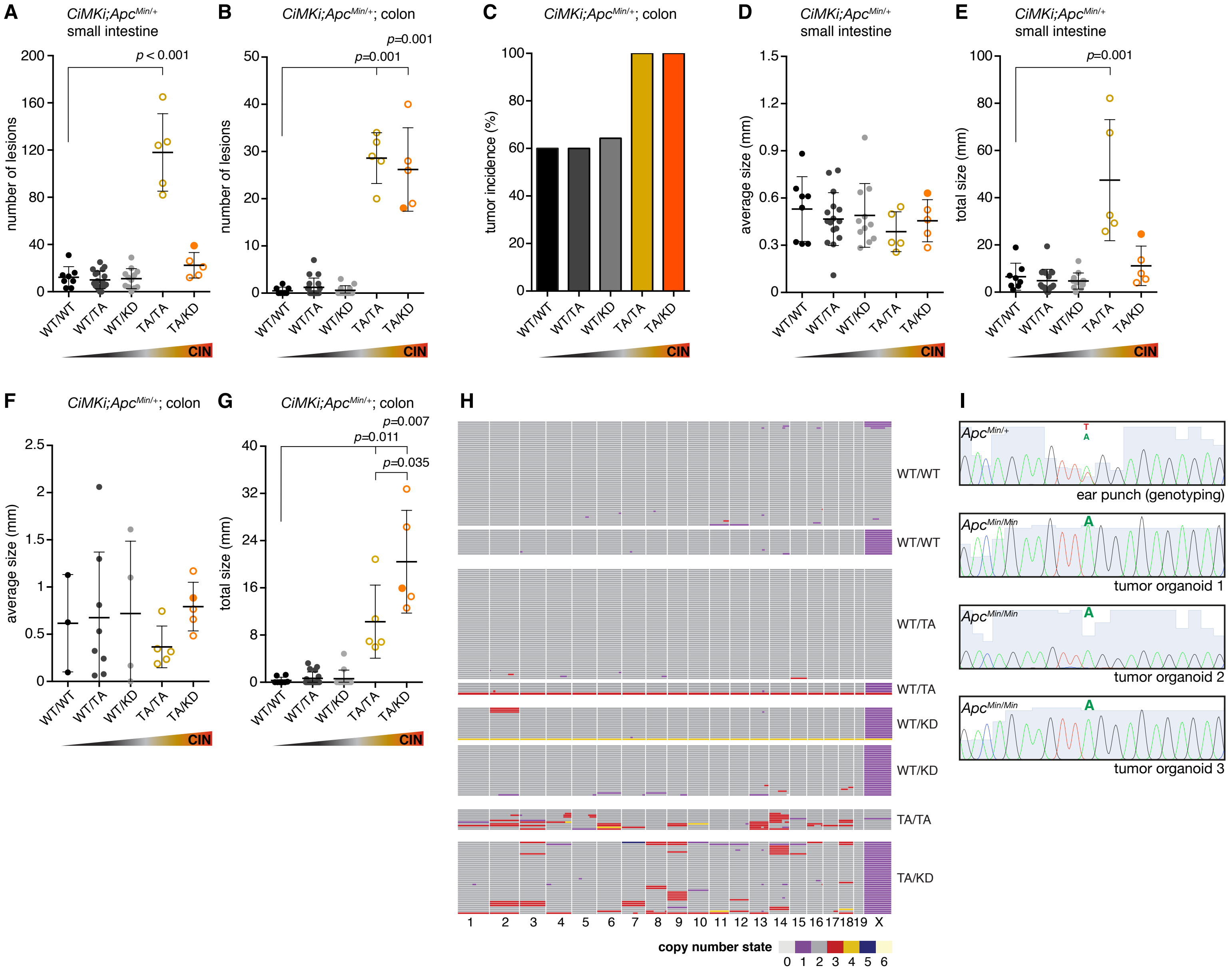

Supplementary Figure 3: CIN differently affects small intestine and colon adenoma formation in *Apc*$^{Min/+}$ mice.
(**A**) Quantification of small intestine adenomas on H&E sections of *CiMKi;Apc*$^{Min/+}$*;VillinCre* mice. Open dots represent mice euthanatized at 6-8 weeks of age, closed dots represent mice euthanatized at 12 weeks of age (n= 8 (WT/WT), n=18 (WT/TA), n=12 (WT/KD), n=5 (TA/TA, TA/KD) mice per group). Data represents mean ± SD, one-tailed Welch's t-test, comparing each group to *CiMKi*$^{WT/WT}$*;Apc*$^{Min/+}$; exact p-values are indicated when <0.05. **(B)** As (A) but for colon adenomas from the same groups of mice. **(C)** Colon adenoma incidence in *CiMKi;Apc*$^{Min/+}$*;VillinCre* mice of the indicated genotypes as in (A, B). **(D)** Average size of small intestine adenoma for each mouse (groups as in (A)) was measured by taking the diameter of the lesion on H&E slides of *CiMKi;Apc*$^{Min/+}$*;VillinCre* mice. Data represents mean ± SD. **(E)** Total adenoma burden in small intestine as the sum of all adenoma diameters per mouse. Data represents mean ± SD, one-tailed Welch's t-test, comparing each group to *CiMKi*$^{WT/WT}$*;Apc*$^{Min/+}$*;VillinCre*; exact p-values are indicated when <0.05. **(F, G)** As (D, E) but for colon adenomas from the same groups of mice. **(H)** scKaryo-seq (bin size 5 MB) (see also Fig. 3H), showing cells of additional examples per genotype. Colors indicate copy number state for a given chromosome. **(I)** Targeted sequencing confirming absence of wild-type *Apc* genomic DNA in adenoma organoids. *Apc*$^{Min/+}$ mouse ear genomic DNA is given as reference. Source data for panels A-G are provided as a Source Data file.

Hoevenaar et al., Supplementary Figure 4

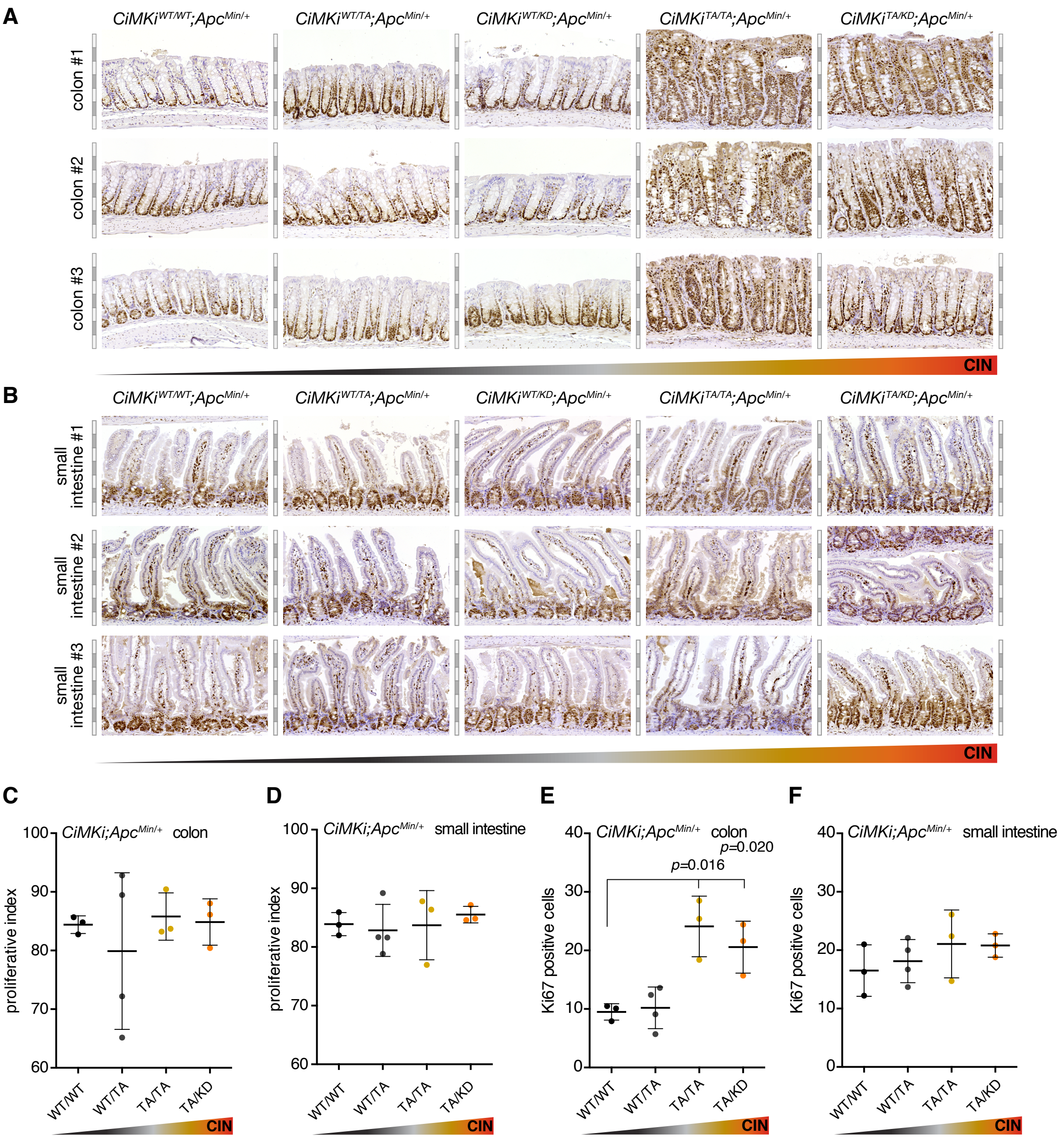

Supplementary Figure 4: Enhanced proliferation in colon but not in small intestine.
(A, B) PCNA staining of tissue sections from colon (A) and small intestine (B) of 4-week old *CiMKi;Apc$^{Min/+}$;VillinCre* mice, as a proxy for proliferative activity, showing increased size and PCNA positivity of colon crypts from *CiMKi$^{TA/TA}$;Apc$^{Min/+}$;VillinCre* and *CiMKi$^{TA/KD}$;Apc$^{Min/+}$;VillinCre* mice. Images are from three mice per genotype. Crypts were selected from similar regions (~2/3 from proximal site). Alternating grey and white scale bars 50 µm. **(C, D)** Proliferative index in colon (C) and small intestine (D) of 4-week old *CiMKi;Apc$^{Min/+}$;VillinCre* mice as determined on ki67 stained tissue sections by calculating the percentage of ki67 positive cells within the proliferative compartment. Data represents mean ± SD of 3 mice (WT/WT, TA/TA, TA/KD), or 4 mice (WT/TA). **(E, F)** Number of ki67 positive cells in proliferative compartment in colon (E) and small intestine (F) of 4-week old *CiMKi;Apc$^{Min/+}$;VillinCre* mice as in (C, D). Data represents mean ± SD, one-tailed Welch's t-test, comparing each group to *CiMKi$^{WT/WT}$;Apc$^{Min/+}$;VillinCre*; exact p-values are indicated when <0.05. Source data for panels C-F are provided as a Source Data file.

Supplementary Table 1: Primers for genotyping and cDNA analysis

Gene	Forward primer	Reverse primer	Expected band size	Sequence primer	Mutation sequence
CiMKi	*CCAAATGGCTAG GGGAGCCACTGA TG*	*GGTGAGGTTGTT TCCAACTGGTAG*	**Mutant 250 bp**	NA	NA
CiMKi (mutation)	GTGTCCTCACCC TGAAAATG	CAAAGCACAGC TGGGCTGTAGA G	**~1000 bp**	CGGATTTTATTTT GAAGGTATTG	T649A ACAA/GCA D637A TTGGA/CT
CiMKi cDNA (mutation)	CCTAGAAGACG CCGATAGCC	GTCTCTGATTGC TTCTGGGGC	**~400 bp**	GATAAGATCATC CGCCTCTATG	T649A ACAA/GCA D637A TTGGA/CT
Apc$^{Min/+}$	*CCGGAGTAAGCA GAGACACAAG*	*CTGTCGTCTGCC ACACAATG*	**~400 bp**	*CATGACTGTTCTTT CACC*	*GACAGAAG TTT/A*
Rosa26-CreER^{T2} (mutant)	GGCAGGAAGCA CTTGCTCTCCC	CCTGATCCTGGC AATTTCG	**~825 bp**	NA	NA
Rosa26-CreER^{T2} (wild-type)	GGCAGGAAGCA CTTGCTCTCCC	GGAGCGGGAGA AATGGATATG	**~650 bp**	NA	NA
Villin-Cre(ER^{T2}*)*	*CAAGCCTGGCTC GACGGCC*	*CCTGATCCTGGC AATTTCG*	**~220 bp**	NA	NA

Supplementary Methods

Cloning of CiMKi targeting vectors

CiMKi alleles were cloned into the pAC targeting vector (based on pFlexible[1]) (kind gift from Jos Jonkers). Three fragments were ligated separately into the vector as follows:

1) Conditional fragment into HindIII-PacI (between loxP sites)

2) 5' Recombination arm into PmeI-AscI (upstream of 5' loxP site)

3) 3' Recombination arm, including either point mutation, into SbfI-NotI (downstream of 3' loxP site).

Details for the three fragments:

1) Conditional fragment

The conditional fragment was obtained by assembling three separate PCR products by ligation into pCDNA3 (see details below):

#1=*HindIII*-(last part of)intron16-*XhoI* (~600 bp)

#2=*XhoI*-**exon17-exon18-exon19-exon20-exon21-exon22**-*BamHI* (~1000 bp)

#3=*BamHI*-intron22(polyAsignal)-*PacI* (~600 bp)

This resulted in the following complete sequence of the conditional fragment (exon17-22 part (PCR product #2) in bold):

*AAGCTT*ATGGCCAGTTTTTCAGTCTCCAAAGGATTTCTCTTTGGAGCCCGGTGTGAGAGT
TGGTCTGTTCTTGCTGTTTCTGTTTCATTATTTGTTTGTTCTTGTTCTCTCCAGTTTCCTCT
GCCTTTCCCTTGTCTTTTAAACAGATGTGTTCAATCTCATGCGCTTCCTCTGCTTCTCCTTT
TAGAAGCAGTAACTAAGCAGAGGATAGGACTCTTTGGGGGTGAGGGAGGCCTTGCGATC
TTATTGTCTGACAGTTCTTGGTGGTGCTTTCCTGGCCTGTGCATGGCAGTGTGCAGCACAC
ACTGACAGGCTAGGCCTGGAATCCTGAACAGTTCACTTCAACTCAAATCACATTGAGTGT
CCTCACCCTGAAAATGATGATGATAATGGGACTTATTTGCTATGCATTGCTATAGAATGG
TACCTGGCCCACAACATTTGCTAAGAAAGTGTAGAATGTATAATAACTAAATATTAATGT
GTTGCCTAATTGAAAGGATAAGCCTGACCTAAAGATTATGAAATGCTTTTTTGTCCCCTG
GCCATAGAATGTTTTTCTTCATCTAACAGAACGGATTTTATTTTGAAG**GTATTGTTCATA**
GTGATCTGAAGCCTGCTAACTTTGTGATAGTGGATGGAATGCTAAAGCTAATTGATT
TTGGGATTGCAAACCAAATGCAGCCAGACACAACAAGCATTGTTAAAGATTCTCAG
GTTGGCACAGTTAACTATATGGCCCCAGAAGCAATCAGAGACATGTCTTCTTCAAG
AGAAAATTCGAAAATCAGAACCAAGGTAAGTCCCAGAAGTGATGTCTGGTCCTTGG
GGTGCATTTTGTACTACATGACTTATGGGAGGACGCCATTTCAGCACATCATCAATC
AGGTCTCTAAACTGCACGCCATAATCAACCCTGCTCATGAGATTGAATTTCCCGAGA
TTTCGGAAAAAGATCTTCGAGACGTGCTAAAGTGCTGTTTAGTGAGGAACCCTAAA
GAGAGGATATCTATCCCTGAGCTCCTCACACATCCGTATGTTCAAATTCAGCCCCAT
CCAGGCAGCCAAATGGCTAGGGGAGCCACTGATGAAATGAAATATGTGTTGGGTCA
ACTTGTTGGTCTGAATTCTCCTAACTCCATCTTGAAAACTGCAAAAACTTTGTATGA
ACGTTATAATTGTGGTGAAGGTCAAGATTCTTCGTCATCCAAGACTTTTGACAAAAA
GAGAGAAAGAAAGTGATGCACAGCTACGTACAAACCAAGAACACTAGATTGTTTCC
TCTGCCATACTCTTGAATCTCTGAGGAAATCTACCAGTTGGAAACAACCTCACCTGG
ATTTTATCAGTTAAAAAAACAAACAAACAAAACTTCAGTAGATTATCCTCAAAAGAA
AGCTGTAAAGTTAACCACTCATAGCACTGTGTATATTAAATTATAGAGTTGTGCTTT
TCTTTTATGCTTTTCTGTAAATCTGCTAATGTTTTACGTTTGGAACAGTGAATGATA
GCTGGAATGTTGAAGAGCTCTGTAAATAAAGCGTCACCACAGTTCCAGAACTGTACA
GTGGTCAGTTTCTTCAATCAAATGTGTTCTTGGCATGATAGCAAAATTTTTAGAAAAACG
GGATTAAGAATAGACCGTAGTAAATAAAGTTTAACAATTAAATTTCCAAAGGATTTAGG
ACTGTAAACAGGCTCACACCTTTAGTCCCAGCACTTGGGAGGCAGAGGCAGGCAGATTT
CTGAGTTCGAGGCCAGCCTGGTCTACAGAGTGAGTTCCAGGACAGCCAGGGCTACACAG
AGAAACCCTGTCTTGGCGGGGAGGGGCCGGGGAGGGGGGGAGATCCAAAGGATGTAGG
AGGCAGAGACAGGTGGATCTCTGTGATTCTGAAGCCAGCCTGGTCTACAGAACTAGTTCT
AGGATAGCCAAGGCTATACAGACACTTTCTCCCCACCACCATCCTGCCCTCAAAAAAATT
GTAAATAAATTTCCTAATTGTGTACAGCCATGATACCATCTATAGTATTTGGTCTGCAAG
TGGCTTTTTCAGTTCTCCCTTTGACTCTTCAAAGTACATATGGGGTTTGGTGTTCTAAATA
TTGTGCTGGAGTTTGTGATTTAATGTCTATAGTTAATACATGCCATTATTGAG*TTAATTAA*

Product #1 was obtained by standard PCR using genomic DNA from 129/Ola-derived IB10 ES cells (kind gift from Hans Clevers). Primers used (*Italic* is overhang, in *Capitals/Italic* the restriction sites):

#1 Forward: *cggcgAAGCTT*ATGGCCAGTTTTTCAG

#1 Reverse: *cgccgCTCGAG*CTTCAAAATAAAATCCGTC

Product #2 was obtained by standard PCR using mouse cDNA (Imagenes, Germany, BC 058851, ID: 30023533). Primers used (*Italic* is overhang, in *Capitals/Italic* the restriction sites):

#2 Forward: *ccggcCTCGAGgcgctactctggtg*GTATTGTTCATAGTG

#2 Reverse: *ccgcgGGATCCgcgctactctggtg*CTGGAACTGTGGTGAC

Product #3 was obtained by standard PCR using genomic DNA from 129/Ola-derived IB10 ES cells (kind gift from Hans Clevers). Primers used (*Italic* is overhang, in *Capitals/Italic* the restriction sites:

#3 Forward: *ccgccGGATCC*AACTGTACAGTGGTC

#3 Reverse: *ccgccgTTAATTAA*CTCAATAATGGCATG

First, products #1 and #2 were ligated by standard methods into pCDNA3 simultaneously. XhoI site was removed using site-directed mutagenesis. Primers used:

(XhoI)loop Forward: GACGGATTTTATTTTGAAGGTATTGTTCATAGTGATC

(XhoI)loop Reverse: GATCACTATGAACAATACCTTCAAAATAAAATCCGTC

Second, product #3 was ligated into the pCDNA containing #1 and #2. BamHI site was removed using site-directed mutagenesis. Primers used:

(BamHI)loop Forward: GCGTCACCACAGTTCCAGAACTGTACAGTGGTCAG

(BamHI)loop Reverse: CTGACCACTGTACAGTTCTGGAACTGTGGTGACGC

This resulted is completion of the conditional fragment. Third, the conditional fragment was digested from pCDNA and ligated into pAC16 (using *HindIII and PacI restriction sites*).

2) 5' Recombination arm

The 5' recombination arm fragment was obtained by standard PCR using genomic DNA (from 129/Ola-derived IB10 ES cells (kind gift from Hans Clevers). Primers used (*Italic* is overhang, in *Capitals/Italic* the restriction sites):

5'arm Forward: c*tagcgGTTTAAAC*TCGAAGGCCTCAACCTCACAGAGATCTTTC

5'arm Reverse: *atcttaGGCGCGCCGGGCCC*TCTCCTCCTATCTGTAGGATG

This resulted in the following complete sequence of the 5' recombination arm:

(*PmeI*-(lastpartof)intron14-**exon15**-intron15-**exon16**-(firstpartof)intron16-*ApaI-AscI* (~2kb):

*GTTTAAAC*TCGAAGGCCTCAACCTCACAGAGATCTTTCTGCTTCTGCCTCTCTCCTGAGTG
CTGAGATTAAAGGTGTGTGCAGCCATGCCTAGCTGTGTGTGGCTTTCCTGTTACCACATT
CTGTGGATAGCTTATCTGTTGTTTGTGCCCCACCTTGTACTTAATATCAAGATGAGATGTT
TGGCTGTTCGGTGATACTAGCTCTGGTGACTAAAACTGAATAGGACCTTTATTCCTTTGCT
GAGTGTTCCTCCTTGGCTATCAGGCTGTTTTATGTTATGGTGATACATGAACAGAGATAA

AGGGCTTATTTAAGTTTTACTGTAATTCTCTAAGCCAGCTAGCTGTAATTAGACTTGCTT
CTGGCTATTTTATTAACTTATAGCAAGTAATTGGAAAGCATCCCATCAGACCCATTTGTA
TAATTCCTGCACACAGTACGCTGAGGCGGGATGATTGCCTGGGCTACATACATGCACTGT
ATAGGCTGCTGCTATGGCTTTGGTGCAGGGCCCTGGCTTTAATCTCTTAACATAAAAACC
ATAAGCAAAAGACAAAATAGGTAGGAGTGTATATTTCCACATGGAGCATGTCTTCCCAT
AAATATTTTCCTTTTCACGCTCCCCTTATTAGATTTTCAGTTATGAGCATAAGGAAGAAGG
TGGGGTAAGAGTGATTGAACAAGAGTGACAGGGAAGAGGATTCGTGCAGGGGAGGGAA
GTGCATGCATGTTCTAGGCACTGAGTGATGGGTGTGCTGGAAGCTGTAAACTGCGTGGG
GGCCTCTTCCTCAGTGCTTTAAGAAATTGATTCATAGGAACATCATTGCTCCTGCCAACCT
AACTCAACTGTGACTTGCGCTGCTTTCCACAAATGAATGTAGTGATGGCTTACAATTACT
GTGATTTTTAAAAATATTCCCTATCAGAGAAATGAATTGGTTGATAGTAGGCACAATGAA
AAGGTGGGAGTTGGTGGGGAGAGGGGTCTAGGAAGTGAACTGTCAAATCACAGCCTTAG
TCATCCTTATCGTCTTCGAATGTCTTTTCCTTGATGTTTTCTCCCTGGATAAGAAAGGCAT
CCCTAGAATTTTGTGGATATAGCAACATCATATTTAAGTTGGTTTTCTTAGACACTGATGT
AGAAAACCTTTGAATTATTTGAATGTCCATTGTTATAGGGGCTGGAAATGGATACTTAGC
TTCTCATGTTGGTATTTCTTTAGGAATATCCTCAGCCTGAGACTGTTAGTGTTAAATGGAA
AGGTACTGCTCCAGTTTTCAGAGGGAGACATGTCCTAAGCTCTTTCTCCACTTTTTATGTA
G**GTGTTTCAGGTATTGAATGAGAAAAAACAGATAAACGCTATCAAATATGTGAACCT
AGAAGACGCCGATAGCCAAACTATTGAGAGCTACCGCAACGAGATAGCGTTTTTGA
ACAAACTACAGCAACACAGTGATAAGATCATCCGCCTCTATGATTA**GTATGAATTCA
TTTTTATTTTAAAAATAAAAGTTTGTTCTTGCCATAATTCTTAGGCAAAGAGTAAATCCTT
AATGACATAATGTGGGCATTTATTGTTTTGTTGTGTCTGTTTATCTTTAATTGCAG**TGAAA
TCACCGAGCAGTACATCTACATGGTAATGGAATGTGGAAACATTGACCTAAATAGT
TGGCTTAAAAAGAAAAAATCCATCAATCCATGGGAACGCAAGAGCTACTGGAAAAA
CATGTTGGAGGCAGTACACATAATCCATCAGCATG**GTATTTTCATATCTCTTCATACA
CGTAAAGTTAAAATAGTTGTTAATTGTGCCATTTTAGAAACATACCCTTAACTGGAAGTT
CATTAGAGGTGAAGGCACTCTTAAGAGTGGTTATACACAGGCTACAGAACACAAACAAG
CACAGGATGTAGAACAGAAATGGCCACATGTACAATGTAAACTTACCCTCCTCTGGTACC
TGGGGATTCCTATCTTCAAGTCCTGAGGATTTGGACATCCTACAGATAGGAGGAGA*GGG
CCCGGCGCGCC*

This fragment was ligated into pAC16 containing the conditional fragment (using *PmeI and AscI restriction sites*, upstream of 5’ loxP site).

3) 3’ Recombination arm

The 3’ recombination arm fragment was obtained by standard PCR using genomic DNA (from 129/Ola-derived IB10 ES cells (kind gift from Hans Clevers)). Primers used (*Italic* is overhang, in *Capitals/Italic* the restriction sites):

3’arm Forward: *ccgccCCTGCAGG*ATGGCCAGTTTTTCAG

3’arm Reverse: *ctagcgGCGGCCGC*CTATTTGCAAATCACAAAG

This fragment was ligated into pAC16 (using *SbfI and NotI restriction sites*, downstream of 3’ loxP site). CiMKi point mutations were introduced using site-directed mutagenesis.

Primers used for T649A mutation:

mMps1-T649A(TA)-F: CAAATGCAGCCAGACACA***GCA***AGCATTGTTAAAGATTC

mMps1-T649A(TA)-R: GAATCTTTAACAATGCTTGCTGTGTCTGGCTGCATTTG

Primers used for D637A mutation:

mMps1-D637A(KD)-F: GAATGCTAAAGCTAATT***GCT***TTTGGGATTGCAAAC

mMps1-D637A(KD)-R: GTTTGCAATCCCAAAAGCAATTAGCTTTAGCATTC

This resulted in the following complete sequence of the 3’ recombination arm (*SbfI*-(lastpartof)intron16-**exon17***-intron17-*NotI* (~2.2kb), containing either T649A or D637A point mutation in exon 17):

*CCTGCAGG*ATGGCCAGTTTTTCAGTCTCCAAAGGATTTTCTCTTTGGAGCCCGGTGTGAGA
GTTGGTCTGTTCTTGCTGTTTTCTGTTTCATTATTTGTTTGTTCTTGTTCTCTCCAGTTTCCT
CTGCCTTTCCCTTGTCTTTTAAACAGATGTGTTCAATCTCATGCGCTTCCTCTGCTTCTCCT
TTTAGAAGCAGTAACTAAGCAGAGGATAGGACTCTTTGGGGGTGAGGGAGGCCTTGCGA
TCTTATTGTCTGACAGTTCTTGGTGGTGCTTTCCTGGCCTGTGCATGGCAGTGTGCAGCAC
ACACTGACAGGCTAGGCCTGGAATCCTGAACAGTTCACTTCAACTCAAATCACATTGAGT
GTCCTCACCCTGAAAATGATGATGATAATGGGACTTATTTGCTATGCATTGCTATAGAAT
GGTACCTGGCCCACAACATTTGCTAAGAAAGTGTAGAATGTATAATAACTAAATATTAAT
GTGTTGCCTAATTGAAAGGATAAGCCTGACCTAAAGATTATGAAATGCTTTTTTGTCCCC
TGGCCATAGAATGTTTTTCTTCATCTAACAGAACGGATTTTATTTTGAAG**GTATTGTTCA**
TAGTGATCTGAAGCCTGCTAACTTTGTGATAGTGGATGGAATGCTAAAGCTAATT*G
CT*TTTGGGATTGCAAACCAAATGCAGCCAGACACA*GCA*AGCATTGTTAAAGATT
CTCAGGTAGGAGTTTTGCTGTCTTGGTTGTATTTTAGTGTTTTGAACCAGGGTTTTGCATC
AGGGTTTTGCATAACCTAGAATGCTCTTGACTTTGATCAGTGGCCTTCAGCTCCTGATCCT
GCTGCCTGTGCATCCCAGGTGTGGGCTTATAGGTGTCAGCCCCGACACCCGACTTCAGGT
AGGATTTTAATGATGGCTGGTTACTACAAGGCTTAGTTCATTTTTATCTGTTAAATATGTT
GCCAATATTATATTTTTACCAACCATGTTATTCCAAAAATTTGAAGTCTTTTTAAAGAATA
GAAACTATGTTTATAAAAGACCATGGTCAAAGCCATGGTCAATTTGATTTATAAAAGCAG
TTCAAGATCAGACAAGTATATTTATGAATTTTGGATGATTTTCTCATAGCTGAGGCAGGG
CAGAGAGTAATTGCACCTTCATGTTCTCCACTGTCCTGTTTCTTTTTCTTACTGCTTAAATT
TGGGAGAAAGTTTTAAGAGAGCCTTATTGGGAATACTGAAGCGTTCCACTCAGCTACGC
GTTAAAAAGGAAATATTTTACTTACTGTTTGGGGGGCGGTGTGCGGATTCCAGATGTAAG
TGTGCCATTGTGGAGGTGTGGGGATGGCTTCGGTAAACCACTTCTCTCCTACTGTGGGCC
CTGGGAGCCAAAGTCAGATTGTGTGTGCAGCAAGCTCTACAGCCCAGCTGTGCTTTGTAG
TAACATTTGCTGTGGTAAATCTCATGAAGCTGAAGTAGTGAGGGGAAAACAGAGCTGAA
AGGTGATGTCGACTGCACCTCGCAGGCTGTGTCCAGGGATGGAGATAAATCAGAAGATA
AATTACCATGCACGTAGAAAGTCATTCTTCTTGACAGCCATTCATTGTTTTTTGTTTCAGA
AGTACAGATGATGAACAGTGAGTGTAGATGAGACTGAAGTTTTCTATGGCAAGGTCTTA
GCAGGCCGACATTTTGTTACCTTAGAACTAAAGGATTTTGCGTATTATCTCCATGCCCAG
CTAGAGAAGCTCTTCTCTGATATAGGTTTCCAACCCATCTTGATCTGCACATGGAGCCGA
AGAATATTGGGAGATAAAGCTAGCTGGTTCCTTTTATTCATGTATTAATTTGTTGCTTGGT
TTATTGAGGGAAGAATATGTTGAATTTATTGGAACATGAAAGTGAATGAAAGGCCAAGT
TCAGAATCCGCCTACGCAGTTGTAAAGACTTAGTACTTAGTACTTAGTACTTAGCACTAG
CTCTCCAGCACAGCTGCAGACAGCACAGTGCTCCCTGTGCTCCAGACGGAGCCCGTTCAT
TCTCAGCCCAGCTCATCTGATTGTACCTGGGATGGGATAGTACATACATTCTTATATTGTT
AGCAGTTATTTGAATTTTTCAAGTCTGTCATTTAAATCATTAGTTATTCAAATTTCCAAGA
ATCTGACATTTACATATTTACAAATCTAGAAAGATATTCTCATTGATTTCTTTGTGATTTG
CAAATAG*GCGGCCGC*
(***GCT* =D637A, *GCA* =T649A)**

These fragments were ligated separately into pAC16 containing the conditional fragment and the 5' recombination arm (using *SbfI and NotI restriction sites*, downstream of 3' loxP site), to obtain the two separate complete targeting vectors containing CiMKi-T649A or CiMKi-D637A.

Fidelity of all PCR products, site-directed mutagenesis steps, and ligation steps were verified by sequencing.

Supplementary Reference

1 Van Der Weyden, L. *et al.* Null and conditional Semaphorin 3B alleles using a flexible puroΔtk LoxP/FRT vector. *Genesis* **41**, 171-178, doi:10.1002/gene.20111 (2005).
