# Supplementary material for: Degree and site of chromosomal instability define its oncogenic potential
Source: Nat Commun. 2020 Mar 20;11:1501. doi: 10.1038/s41467-020-15279-9 (PMC7083897; doi:10.1038/s41467-020-15279-9)
Supplement: Supplementary file 3 — Description of Additional Supplementary Files [file 41467_2020_15279_MOESM3_ESM.pdf]

Description of Additional Supplementary Files

Supplementary Movie 1: Missegregation rate in *CiMKi;Rosa26-CreER^{T2}* MEFs. Time lapse imaging of *CiMKi$^{WT/WT}$;R26CreER^{T2}* immortalized MEFs expressing H2B-mNeon, 56 hours after 4-OHT addition.

Supplementary Movie 2: Increased missegregation rate in *CiMKi;Rosa26-CreER^{T2}* MEFs. Time lapse imaging of *CiMKi$^{KD/KD}$;R26CreER^{T2}* immortalized MEFs expressing H2B-mNeon, 56 hours after 4-OHT addition.

Supplementary Movie 3: Missegregation rate in *CiMKi;Apc$^{Min/+}$;Villin-Cre* colon adenoma organoids. Time lapse imaging of *CiMKi$^{WT/WT}$;Apc$^{Min/+}$;Villin-Cre* colon adenoma organoids. Color dept-coding (purple is bottom of organoid, red is top) was used to identify the position of the cells, left panel) and maximum projections are depicted in the right panel.

Supplementary Movie 4: Increased missegregation rate in *CiMKi;Apc$^{Min/+}$;Villin-Cre* colon adenoma organoids. Time lapse imaging of *CiMKi$^{TA/TA}$;Apc$^{Min/+}$;Villin-Cre* colon adenoma organoids. Color dept-coding (purple is bottom of organoid, red is top) was used to identify the position of the cells, left panel) and maximum projections are depicted in the right panel.
