# Supplementary material for: Degree and site of chromosomal instability define its oncogenic potential
Source: Nat Commun. 2020 Mar 20;11:1501. doi: 10.1038/s41467-020-15279-9 (PMC7083897; doi:10.1038/s41467-020-15279-9)
Supplement: Supplementary file 8 — Reporting Summary [file 41467_2020_15279_MOESM8_ESM.pdf]

nature research

Corresponding author(s): Nannette Jelluma

Last updated by author(s): Jan 7, 2020

Reporting Summary

Nature Research wishes to improve the reproducibility of the work that we publish. This form provides structure for consistency and transparency in reporting. For further information on Nature Research policies, see Authors & Referees and the Editorial Policy Checklist.

Statistics

For all statistical analyses, confirm that the following items are present in the figure legend, table legend, main text, or Methods section.

n/a	Confirmed	
☐	☒	The exact sample size (*n*) for each experimental group/condition, given as a discrete number and unit of measurement
☐	☒	A statement on whether measurements were taken from distinct samples or whether the same sample was measured repeatedly
☐	☒	The statistical test(s) used AND whether they are one- or two-sided<br>*Only common tests should be described solely by name; describe more complex techniques in the Methods section.*
☒	☐	A description of all covariates tested
☒	☐	A description of any assumptions or corrections, such as tests of normality and adjustment for multiple comparisons
☐	☒	A full description of the statistical parameters including central tendency (e.g. means) or other basic estimates (e.g. regression coefficient) AND variation (e.g. standard deviation) or associated estimates of uncertainty (e.g. confidence intervals)
☒	☐	For null hypothesis testing, the test statistic (e.g. *F*, *t*, *r*) with confidence intervals, effect sizes, degrees of freedom and *P* value noted<br>*Give P values as exact values whenever suitable.*
☒	☐	For Bayesian analysis, information on the choice of priors and Markov chain Monte Carlo settings
☒	☐	For hierarchical and complex designs, identification of the appropriate level for tests and full reporting of outcomes
☒	☐	Estimates of effect sizes (e.g. Cohen's *d*, Pearson's *r*), indicating how they were calculated

Our web collection on statistics for biologists contains articles on many of the points above.

Software and code

Policy information about availability of computer code

Data collection	Fluorescent images were collected using SoftWorx 6.0 software (Applied Precision/GE Healthcare), Nikon Imaging Software (NIS-Elements AR 5.51.00), and Cell-M software (Olympus). Library preparation was performed using a Bravo Automated Liquid Handling Platform (Agilent Technologies, Santa Clara, CA, USA). Clusters for sequencing were generated on the cBot (Illumina). Single-end 75 bp sequencing was performed on an Illumina NextSeq 500 (Illumina, San Diego, CA, USA).
Data analysis	Prism 6 was used for statistical analysis and graphical data presentation (https://www.graphpad.com/). ImageJ 1.49b Java 1.6.0_24 [64-bit] and ImageJ 1.49v Java 1.6.0_65 [64-bit] were used to analyze image data. Previously published custom made Fiji-macros were used to generate organoid time-lapse movies from XYZT imaging data sets, and for detailed scoring of mitotic events in the time-lapse movies (Verissimo et al., eLife, 2016), and for quantification of immunofluorescent images (Saurin et al., Nature Communications, 2011). NDP.view2 Software from Hamamatsu was used for analyses of scanned slides. Raw sequencing data were demultiplexed based on library-specific barcodes and converted to fastq format using standard Illumina software (bcl2fastq version 1.8.4). Analysis of single-cell sequencing data was performed with the AneuFinder1.6.0 pipeline (Bakker et al., Genome Biology, 2016), Python 2.7.5 and Burrows Wheeler Aligner 0.7.12.

For manuscripts utilizing custom algorithms or software that are central to the research but not yet described in published literature, software must be made available to editors/reviewers. We strongly encourage code deposition in a community repository (e.g. GitHub). See the Nature Research guidelines for submitting code & software for further information.

Data

Policy information about availability of data

All manuscripts must include a data availability statement. This statement should provide the following information, where applicable:

- Accession codes, unique identifiers, or web links for publicly available datasets
- A list of figures that have associated raw data
- A description of any restrictions on data availability

The scKaryo-seq data have been deposited in the European Nucleotide Archive database under the accession code PRJEB31573 [https://www.ebi.ac.uk/ena/data/

view/PRJEB31573]. All the other data supporting the findings of this study are available within the article and its supplementary information files and from the corresponding author upon reasonable request. Source data for Figures 1C-E; 2C, D; 3C, F, G; 4A-D; Supplementary Figures 1A, B; E-G; 2A-L; 3A-G; 4C-F and a reporting summary for this article are available as Supplementary Information files.

Field-specific reporting

Please select the one below that is the best fit for your research. If you are not sure, read the appropriate sections before making your selection.

☒ Life sciences ☐ Behavioural & social sciences ☐ Ecological, evolutionary & environmental sciences

For a reference copy of the document with all sections, see nature.com/documents/nr-reporting-summary-flat.pdf

Life sciences study design

All studies must disclose on these points even when the disclosure is negative.

Sample size	For in vivo studies, sample sizes were predetermined using power analysis, using 80% power and 95% confidence. In other experiments, sample sizes were not predetermined. For these experiments, sample sizes were chosen based on experience from previous experiments to reach statistically relevant results. Sample size and number of independent experiments are stated in the figure legends.
Data exclusions	No data were excluded from the analyses in this study
Replication	All in vitro experiments were reliably reproduced at least two times. The number of replicates are indicates in the figures or in the legends for each experiment. In vivo experiments were performed once, using group sizes predetermined as stated above.
Randomization	Mice were not randomized but allocated into experimental groups based on genotype.
Blinding	Investigators were blinded to group allocation for data processing, pathological analyses). Mouse, organoid, and cell handling was not blinded, as this was done by the same investigator who set up the experiment.

Reporting for specific materials, systems and methods

We require information from authors about some types of materials, experimental systems and methods used in many studies. Here, indicate whether each material, system or method listed is relevant to your study. If you are not sure if a list item applies to your research, read the appropriate section before selecting a response.

Materials & experimental systems

n/a	Involved in the study	
☐	☒	Antibodies
☐	☒	Eukaryotic cell lines
☒	☐	Palaeontology
☐	☒	Animals and other organisms
☒	☐	Human research participants
☒	☐	Clinical data

Methods

n/a	Involved in the study	
☒	☐	ChIP-seq
☒	☐	Flow cytometry
☒	☐	MRI-based neuroimaging

Antibodies

Antibodies used	anti-Ki-67 (ThermoFisher, RM-9106, Clone SP6)<br>anti-ß-catenin (BD Transduction Laboratories, Clone 14/Beta-Catenin 610154)<br>anti-Mad1 (Santa Cruz sc67337),<br>anti-Centromere Protein (ACA) (Antibodies Incorporated 15-234-0001)<br>anti-ESK (Santa Cruz sc-541)<br>anti-alpha-Tubulin (Sigma T5168)<br>anti-phospho-Histone H3 (Ser10) (Millipore 06-570)<br>anti-PCNA (PC10) (Millipore MAB424)<br>anti-Rabbit Envision-HRP (DAKO)<br>anti-Mouse Envision-HRP (DAKO)
Validation	For each primary antibody used in this study, validations and applications are stated on the manufacturer's websites:<br>ki67: Species Reactivity: Human. Others not tested. Recommended for Immunohistochemistry (Formalin/paraffin).<br>ß-catenin: Reactivity Human (QC Testing) Mouse, Rat, Dog, Chicken (Tested in Development). Application: Western blot (Routinely Tested), Immunohistochemistry, Immunoprecipitation, Immunofluorescence (Tested During Development).<br>Mad1: MAD1(M-300) is recommended for detection of MAD1 of mouse,rat and, to a lesser extent,human origin by Western Blotting, immunoprecipitation, immunofluorescence, and solid phaseELISA.<br>ACA: Applications: Immunocytochemistry, Immunogen: Anti-Centromere Antibodies (derived from human CREST patient serum) are useful as controls in ANA diagnostics and also react with other species including mouse, rat, and hamster.

ESK: Esk(C-20) is recommended for detection of Esk of mouse origin by Esk(C -20) by Western Blotting , immunofluorescence, and solid phase ELISA.
alpha-Tubulin: Applications: IF, RIA, WB. Reactivity: chicken, kangaroo rat, sea urchin, rat, Chlamydomonas, bovine, human, African green monkey, mouse.
phospho-Histone H3 (Ser10): validated in ICC, IP & WB.
PCNA: validated in ELISA, FC,IHC(P), IP, WB

Eukaryotic cell lines

Policy information about cell lines

Cell line source(s)	Mouse embryonic fibroblasts (MEFs) and intestinal organoids were isolated from mice in-house. In-house 129/Ola-derived IB10 ES cells were a kind gift from H. Clevers.
Authentication	N/A for MEFs and organoids. IB10 ES cells were not authenticated by us.
Mycoplasma contamination	Part of the used MEF cell lines and organoids were tested negatively for mycoplasma. Part of the used MEF cell lines and organoids were not tested for mycoplasm.
Commonly misidentified lines (See ICLAC register)	No commonly misidentified lines were used.

Animals and other organisms

Policy information about studies involving animals; ARRIVE guidelines recommended for reporting animal research

Laboratory animals	Genetically modified mice (Mus Musculus) strains used in this study include: ACTB:FLPe (B6.Cg-Tg(ACTFLPe)9205Dym/J, stock number 005703), Rosa26-CreERT2 (B6.129Gt(ROSA)26Sortm1(cre/ERT2)Tyj/J, stock number 008463), and ApcMin/+ (C57BL/6J-ApcMin/J, stock number 002020) and were purchased from JAX® mice. Villin-Cre mice we a gift from S. van Mil (originated from JAX® mice (B6.Cg-Tg(Vil1-cre)997Gum/J, stock number 004586). Villin-creERT2 mice were a gift from J. van Rheenen. CiMKi mice were generated under license of UMCU (DEC 2010.I.02.026). All mice were maintained in C57BL/6 background. For experiments males and females from the age of 4 weeks to 8 months were used.
Wild animals	The study did not involve wild animals.
Field-collected samples	The study did not involve samples collected from the field.
Ethics oversight	Animal experiments were approved by the Animal Experimental Committee Utrecht (DEC Utrecht) and/or the Dutch Central Authority for Scientific Procedures on Animals (CCD), all according to the Directive 2010-63-EU.

Note that full information on the approval of the study protocol must also be provided in the manuscript.
